# Soil Functional Operating Range Linked to Microbial Biodiversity and Community Composition Using Denitrifiers as Model Guild

**DOI:** 10.1371/journal.pone.0051962

**Published:** 2012-12-20

**Authors:** Sara Hallin, Allana Welsh, John Stenström, Stephanie Hallet, Karin Enwall, David Bru, Laurent Philippot

**Affiliations:** 1 Swedish University of Agricultural Sciences, Department of Microbiology, Uppsala, Sweden; 2 INRA, UMR Agroecology, Dijon, France; Jyväskylä University, Finland

## Abstract

Soil microorganisms are key players in biogeochemical cycles. Yet, there is no consistent view on the significance of microbial biodiversity for soil ecosystem functioning. According to the insurance hypothesis, declines in ecosystem functioning due to reduced biodiversity are more likely to occur under fluctuating, extreme or rapidly changing environmental conditions. Here, we compare the functional operating range, a new concept defined as the complete range of environmental conditions under which soil microbial communities are able to maintain their functions, between four naturally assembled soil communities from a long-term fertilization experiment. A functional trait approach was adopted with denitrifiers involved in nitrogen cycling as our model soil community. Using short-term temperature and salt gradients, we show that the functional operating range was broader and process rates were higher when the soil community was phylogenetically more diverse. However, key bacterial genotypes played an important role for maintaining denitrification as an ecosystem functioning under certain conditions.

## Introduction

Rapid decrease in biodiversity due to human activities has lead to a large body of research focusing on the relationship between biodiversity and ecosystem functioning [1]. Ironically, the significance of microbial biodiversity for ecosystem functioning remains largely unknown even though microorganisms are key players in the biogeochemical cycles, which all relate to several ecosystem functions such as nutrient cycling, carbon cycling, climate regulation and plant productivity [2]. Studies using natural microbial diversity gradients [3], [4] or manipulating microbial diversity in microcosms, either by assembling communities or diluting natural communities [5], [6], [7], have not resulted in a consistent view on the link between microbial biodiversity and ecosystem functioning. For example, Wertz et al. [7] reported that ecosystem functioning was largely unaffected by biodiversity erosion, whereas Maherali and Klironomas [8] found a positive relationship between fungal diversity and ecosystem functioning when diversity resulted in increased functional trait richness. Results by Wittebolle et al. [9] suggest that evenness rather than richness as a measure of bacterial biodiversity favors a rapid response to selective stress and thereby plays a role for functionality.

The positive effects of biodiversity on ecosystem functioning are attributed to complementarities among species that would enhance resource use or simply to the increased probability of finding a few high-performing key species within the community [10]. Both these mechanisms would lead to better community performance and thereby preservation of ecosystem functioning. The idea that increased biodiversity insures ecosystems against declines in functioning [11] was further developed by Yachi and Loreau [12] who tested the insurance hypothesis and demonstrated that higher diversity reduces the temporal variance of ecosystem processes. They also showed that the way the different species respond to environmental change affect the level of species richness at which the ecosystem process saturates. Within this paradigm, biodiversity becomes most important under fluctuating or rapidly changing environmental conditions. Accordingly, declines in ecosystem functioning and in corresponding microbial processes due to low or reduced biodiversity are more likely to be observed in variable or extreme, rather than under stable or optimal environmental conditions. Yet, most experimental studies have tested the relationships between microbial biodiversity and ecosystem functioning by measuring microbial process rates under optimal conditions, e.g. [5], [7].

Here, we used the insurance hypothesis to address the role of microbial biodiversity for ecosystem functioning using naturally assembled soil microbial communities as our model system. Maherali and Klironomas [8] stressed the importance of allowing community assembly that reflects realistic, non-random ecological processes when testing mechanisms regulating the relationship between biodiversity and ecosystem function. The trait complementarity that would maintain ecosystem functioning in artificially assembled communities may not reflect that found in a naturally assembled community if these traits do not also support coexistence among the same community members [13]. The soils selected for this study had developed differences in their microbial community diversity and composition in response to more than 50-years of different fertilization regimes [14]. We hypothesized that soil communities with the highest genotypic dissimilarity also have highest functional dissimilarity or complementarity, and thereby higher tolerances to extreme conditions. This would result in a broader functional operating range, which we define as the range of environmental conditions under which a community or ecosystem is able to maintain its functions. Rather than describing the diversity and functioning of all microorganisms, we targeted a subset that performs a specific function, the denitrifying bacteria. Under anaerobic conditions denitrifiers respire using nitrate, which is then reduced to nitrogen gas in four consecutive steps. Because of their widespread representation across the bacterial domain, physiological breath and life style features, and high diversity in most ecosystems, denitrifiers are considered to be a good model community for investigating the functional significance of microbial diversity [9], [15]. They are crucial for major ecosystem services such as nitrogen cycling and climate regulation through emissions of the greenhouse gas N_2_O. In the present study, denitrification was used as a proxy for ecosystem functioning and denitrification rates were monitored over broad temperature and salt gradients. Denitrifier biodiversity in terms of genotypic dissimilarities was assessed using traditional biodiversity indices and phylogenetic diversity metrics in order to more fully evaluate the link between the denitrification operating range and the biodiversity of the denitrifying community.

## Materials and Methods

### Site and Soil Sampling

To sample naturally assembled denitrifier communities, we used soil from the Ultuna long-term soil organic matter experiment in Uppsala, Sweden established in 1956 from the following treatments: unfertilized bare fallow (A), planted with maize and unfertilized (B), fertilized with calcium nitrate (C) and fertilized with cattle manure (J). The set up is a block design with three independent replicate blocks for each soil treatment partitioned into 2 by 2 m plots. Composite samples of ten soil cores (2-cm diameter, 20-cm depth) were taken from each treatment plot (n = 12) in between rows in October 2007 after harvest, sieved (4-mm mesh) and stored at −20°C. Access to the site and data on soil properties were provided by The Department of Soil and Environment, Swedish University of Agricultural Sciences, Uppsala (see Supporting Information, Table S1), and were similar to soil properties determined previously [14]. Soil conductance was measured using an Oakton hand-held pH/mV meter (Cole-Parmer Instrument Co., Vernon Hills, Illinois, USA).

### Denitrification Activity under Temperature and Salt Gradients

The functional operating range for the microbial process denitrification, used as a proxy for ecosystem functioning, was assessed by measuring potential denitrification activity across temperature and salt gradients for each of the three field replicates from each soil community according to Pell et al [16]. Briefly, 8 ml of substrate (1 mM glucose and 1 mM KNO_3_) were added to 8 g soil in flasks that were sealed and then purged five times, by evacuating the ambient air and filling with N_2_. Acetylene was added to reach 0.1 atm partial pressure to block N_2_O conversion to N_2_. The soils were then incubated under agitation at 2, 10, 20, 30, 37, 45, 53, and 60°C. Another set of soil microcosms were set-up similarly to assess denitrification activity in a salt gradient created by addition of NaCl to a final concentration of 0, 0.5, 0.75, 1, 1.5, 2, 3, and 4% w/v and then incubated under agitation at 30°C. For both the temperature and salt gradient experiments, gas samples were collected every half hour during 3 hours of incubation. Nitrous oxide in the gas samples was analyzed on a gas chromatograph (model CP 9000; Chrompack, Rotterdam, The Netherlands) equipped with a ^63^Ni electron capture detector. The amount of N_2_O produced was corrected for the amount dissolved in the liquid at each test temperature [17], [18].

The denitrification rates were calculated from linear regression of the N_2_O produced during incubation using the statistical software R (v 2.9.2; www.R-project.org). To characterize the functional operating range of each soil community, the data on effects of temperature and salt concentration on the denitrification activity were modeled using the statistical software SigmaPlot 12 (Systat Software Inc., San Jose, California, USA). Briefly, the potential denitrification rates under the temperature gradient were fitted with the Gaussian function:
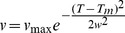
where *v* is the rate obtained at the temperature T, *v_max_* the maximum rate obtained at the temperature *T_m_*, and *w* multiplied by 2.355 gives the full width at *v_max_*/2. Modeled rates according to this equation gave good fits (Fig. S1a) with adjusted *r^2^* values between 0.879 and 0.996 for all the treatments and replicates (n = 12; Table S2). The Gaussian model has previously been shown to be the best to model temperature dependence of heterotrophic soil respiration [19].

Potential denitrification rates under the salt concentration gradient were fitted with a power equation (Fig. S1b):
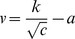
where *c* is the salt concentration, and *k* the slope and *a* the intercept of a plot of *v* against *c* ^−0.5^.

According to this equation, the denitrification rate is predicted to be completely inhibited when *c* = (*k*/*a*)^2^, and to start decreasing from the uninhibited rate *v_0_* at the threshold concentration *c* = [*k*/(*v_0_*+ *a*)]^2^. Fits of the modeled rates (Fig. S1b) had *r^2^* values between 0.938 and 1.000 (Table S3).

The modeled rates were normalized to the maximum rate for each soil community replicate. To test for differences in functional operating range between the communities, the model parameters representing the width of the denitrification rate curves under the temperature gradient and the optimal temperature for denitrification activity were used together with salt concentrations when inhibition of the denitrification activity began and ceased, calculated from the power equation. An analysis of variance (ANOVA) was carried out followed by a Tukey’s HSD test using R at *P*<0.1.

### Molecular Analysis of Genotypic Dissimilarity of Denitrifier Communities

We used the nucleotide variation in the *nos*Z gene encoding the nitrous oxide reductase catalyzing the last step of the denitrification pathway as a molecular marker of the genotypic dissimilarity and phylogenetic diversity of the denitrifier community. The *nosZ* gene is the molecular marker among denitrifiers that is most congruent with taxa affiliation [20] and thus corresponds to genetic distance between community members. This gene is frequently used to characterize environmental denitrifier communities in terms of composition and abundance, e.g. [14], [21], [22], [23], [24].

DNA was extracted in duplicate from 250 mg of soil from each of the soil replicates using the ISO 11063 soil DNA extraction method [25]. Soil samples were homogenized in 1 ml extraction buffer for 30 s at 1600 rpm in a mini bead-beater cell disrupter (Micro-Dismembrator; S. B. Braun Biotech International). Samples were centrifuged (14 000 g for 5 min at 4°C) to eliminate soil debris and nucleic acids precipitated using ice-cold ethanol. Nucleic acids were purified using both PVPP and sepharose 4B columns. The duplicate extracts were pooled and the *nos*Z gene was amplified using primers nosZ-F and nosZ-R [24] slightly modified by Mounier et al. [26] according to the conditions described by the same authors. To minimize PCR bias, three independent PCRs were performed and pooled for each soil sample.

We used cloning followed by restriction fragment length polymorphisms (RFLP) analysis and sequencing to assess denitrifier diversity. The *nosZ* PCR products from each soil sample (n = 12) were gel purified using the MinElute purification kit (Qiagen, France) and cloned using the pGEM-T Easy Vector System (Promega, France). Approximately 100 clones from each of the 12 libraries were randomly selected for amplification using the T7 and SP6 vector primers. The PCR products were then digested with the restriction endonuclease *Alu*I as previously described [26]. *Alu*I was selected after being tested *in silico* on a diverse set of *nosZ* fragments downloaded from public databases. The restriction fragments were resolved by electrophoresis in a 3% high-resolution agarose gel (MP Biomedicals, France) and clones were grouped according to their restriction profile. Randomly selected clones from each RFLP group were sequenced by Beckman (Beckman Coulter, UK) using the vector primer T7. In addition to phylogenetic analysis, the *nosZ* sequences were used to verify the RFLP restriction profiles and ensure representative sampling of the soil treatments. The sequences were analyzed for restriction sites using Geneious v 5.0.4 (Biomatter Ltd, Auckland, New Zealand). To display how the RFLP groups correspond to the *nosZ* phylogeny, a maximum likelihood (ML) phylogenetic analyses (see below) of representative sequences from each RFLP group and similar sequences from pure cultures and other soil clones retrieved from Genbank was performed. The 400 *nosZ* sequences were deposited in Genbank under accession numbers JF310276 to JF310675.

### Analyses of Diversity and Community Composition

To explore differences in genotypic dissimilarities among soil communities, shared RFLP groups were visualized in a Venn diagram using the software mothur [27]. Chao and Shannon’s diversity (*H’*) indices for each soil community were calculated based on RFLP patterns [27]. Differences in diversity revealed by RFLP among and between communities were tested for significance using analyses of molecular variance (AMOVA; [28] using Arlequin 3.01 (Computational and Molecular Population Genetics Lab CMPG, Geneva, Switzerland).

The phylogenetic diversity metric and phylogeny based community composition were calculated using the 90, 99, 77 and 134 sequences representing the RFLP groups from soil communities A, B, C and J, respectively. *nosZ* nucleotide sequences were translated into protein residues, aligned using CLUSTAL X in Geneious v 5.5 (Biomatters Ltd. Auckland, New Zealand) and then back-translated into aligned nucleotides using MacClade v 4.05 (Sinauer Associates, Sunderland MA, USA). Maximum likelihood (ML) phylogenetic analyses for this dataset of 400 sequences of 708 bp were completed using the RAxML-HPC Blackbox (v 7.2.6) program [29] on the CIPRES cluster (www.phylo.org). The ML tree was displayed and annotated by treatment using the Interactive Tree OF Life (iTOL) [30]). From this ML tree, phylogenetic diversity (PD) [31] was calculated for each replicate sample and then by soil community. The PD metric measures the divergences among *nosZ* genotypes calculated as the sum of branch lengths separating genotypes in the phylogeny. Thus, the total amount of phylogenetic distance among genotypes in a specific community is estimated, which is influenced both by how related genotypes are to each other on average and how many that are present. If we assume that closely related genotypes are more similar and have similar niches, PD integrates several aspects of biodiversity. In addition to PD, net relatedness index (NRI) testing basal relationships and nearest taxon index (NTI) testing relatedness at the tips of the tree were calculated to gauge the degree of phylogenetic clustering or overdispersion for sequences by replicate and soil community across the phylogeny [32]. Negative values indicate higher than expected phylogenetic diversity in the assemblage given the richness of genotypes in the community, whereas positive values suggests environmental filtering. The PD, NRI and NTI metrics were calculated using the picante package in R [33]. Parametric and non-parametric tests for significant differences among soil communities and pairwise correlation analyses among these measures and modeled rate parameters were completed using R.

Phylogeny-based community composition among the 12 soil communities were tested using unweighted UniFrac analysis, which calculates the unique fraction of shared branches on the ML tree pairwise for each replicate based on the presence or absence of shared lineages in the tree [34]. The UniFrac distance matrix (Bray-Curtis) was analyzed by non-metric multidimensional scaling (NMS) using the standard metaMDS function in the vegan package in R. This includes a maximum of 20 random starts in search of a stable solution, i.e. when two similar configurations with minimum stress are found. The envfit function in the vegan package was used to test significant correlations of each diversity metric and modeled rate parameter with the ordination. Mantel test was used to determine if the UniFrac matrix and matrices of diversity indices metrics and denitrification rate model parameters were significantly correlated using 999 permutations.

## Results

### Functional Operating Range of Soil Denitrification

**Figure 1:**
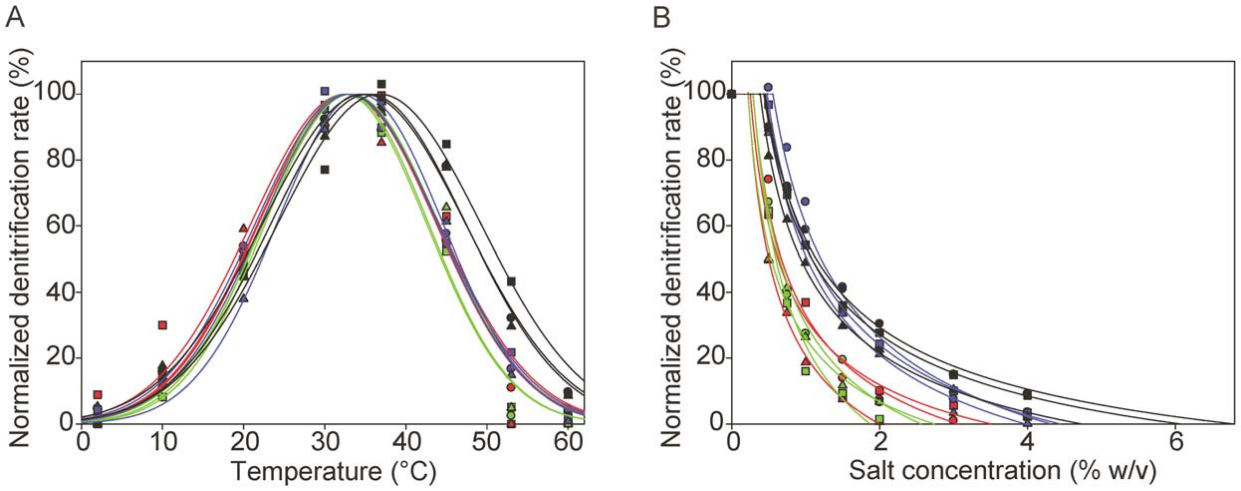
Normalized denitrification rates at different temperatures and salt concentrations. The rates are normalized by percent of the maximum rate within each soil community replicate and modeled using a Gaussian function and a power equation for temperature (a) and salt concentration (b) gradients, respectively. The fitted curves and data points are colored by community and soil treatment: Red, community A, fallow; Green, community B, unfertilized; Blue, community C, nitrate fertilized; and Black, community J, cattle manure fertilized. Each field replicate within a treatment/community is represented by different symbols.

Significant differences in denitrification activity were observed between the soil communities. Community J had the highest activity in both the temperature and salt gradient experiments with lower rates in decreasing order for C, B, and A coincident with significant decreases in soil carbon and nitrogen content (Figs. S1a and b; Table S1). To test for differences in functional operating range between the soil communities, parameters obtained from the modeled normalized denitrification rates were compared. Tukey’s HSD indicated that the model parameter representing the width (*w*) of the denitrification rate curves in the temperature gradient was broader in community J (Fig. 1a; Table 1; *P*<0.1) than in the other communities, for which no significant differences were observed. Similarly, the optimum temperature for denitrification (*T_m_*) was not different for communities A, B, and C, but was significantly higher for community J.

Extrapolated values for salt concentrations when inhibition of the denitrification activity (SAI) began showed that communities A and B were significantly different from C and J, which had higher salt tolerance (Fig. 1b; Table 1; *P*<0.1). However, comparison of the extrapolated salt concentrations when the denitrification rates were completely inhibited (SA0) indicated significant differences between community J and the other communities, with J being active up to 5.9% salt, whereas communities A, B, and C ceased denitrification activity at 2.9, 2.4, and 4.2% respectively.

**Table 1:** Model parameters representing the functional operating range for potential denitrification rates in assembled soil communities under temperature and salt concentration gradients modeled using a Gaussian function and power equation, respectively.

Soil community (treatment)	Temperature gradient†		Salt gradient§	
	*T_m_*	*w*	SAI	SA0
A (Fallow)	32.7(0.2)^a^	11.2(0.3)^a^	0.254(0.032)^a^	2.86(0.78)^ab^
B (Unfertilized)	32.8(0.4)^a^	10.4(0.4)^a^	0.267(0.040)^a^	2.39(0.46)^a^
C (Nitrate fertilized)	33.4(0.9)^a^	10.8(0.5)^a^	0.499(0.053)^b^	4.24(0.24)^b^
J (Cattle manure fertilized)	35.6(1.0)^b^	12.4(0.3)^b^	0.425(0.035)^b^	5.87(1.04)^c^

Mean values of three field replicates of the soil communities are shown with standard deviations (±SD). Values followed by the same letter indicate treatments without significant differences (*p*<0.1).

† Gaussian function: Denitrification rate = *V_max_* * e^–(*T-Tm*)^2^^/(2*w^2^^)^ where *V_ma_*_x_ = maximum denitrification rate, *T* = tested temperature, *T_m_* = optimum temperature and *w* = measure of the width of the curve.^^

§ Power equation: Denitrification rate = *k*/√(*c*) - *a*, where *k* = slope, *c* = % salt concentration and *a* = intercept. From the equation, the salt concentration when denitrification begins to be inhibited (SAI) and when the rate reaches zero (SA0) were calculated.

### Denitrifier Community Diversity Measures and Genotypic Dissimilarities

**Figure 2:**
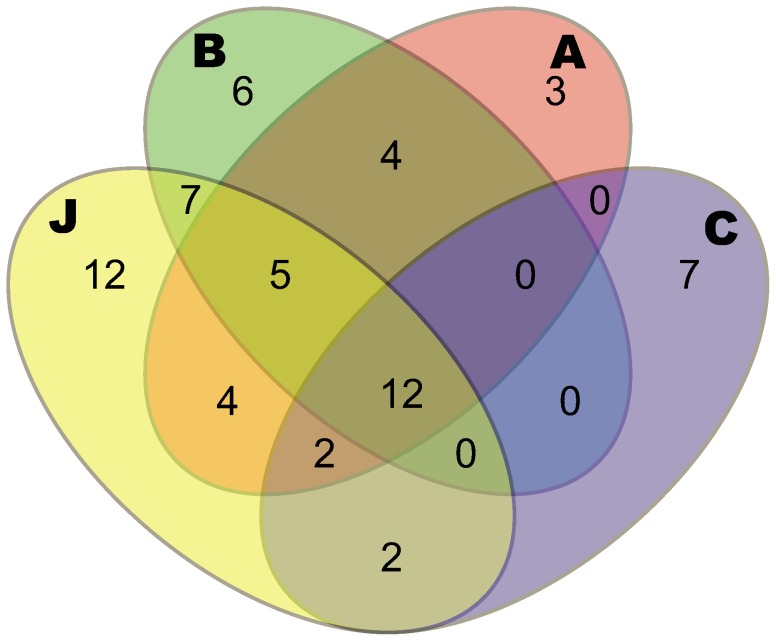
Venn diagram representing shared and unique restriction fragment length polymorphisms (RFLP) groups for denitrifying communities. The groups were detected by screening ∼100 *nosZ* gene clones retrived from soil communities A–C and J. Community A had 30, B had 34, C had 23 and J had 44 of the 64 groups in total.

**Figure 3:**
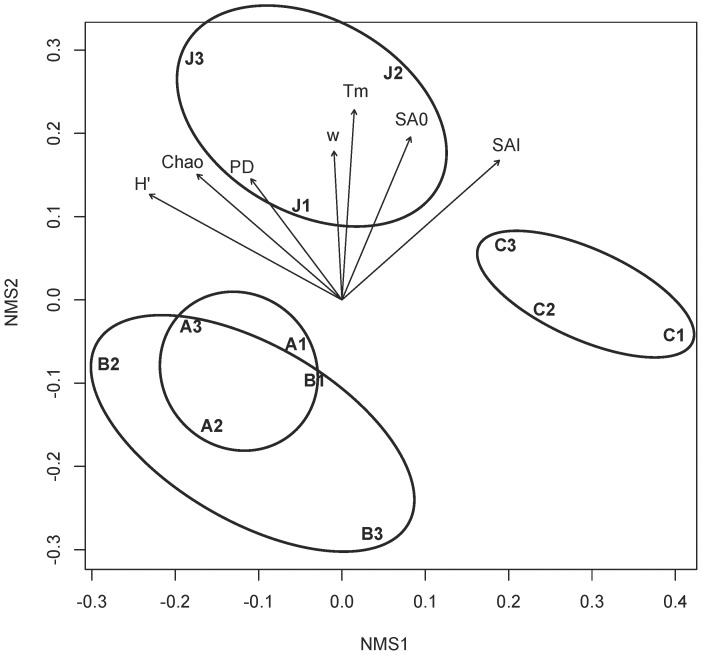
Non-metric multidimensional scaling of phylogeny-based composition of denitrifying communities. The distance matrix was based on pairwise differences in the unique fraction of branches shared (UniFrac) for each soil community replicate in the maximum likelihood phylogentic tree of 400 *nos*Z gene sequences. Vectors indicate diversity metrics and modeled rate parameters that were significantly correlated with the ordination (*P*<0.1): Chao richness index, Shannon’s diversity index (*H´*), phylogenetic diversity (PD), width of the modeled temperature gradient curve (*w*), denitrifiction rate at temperature optimum (*T_m_*), salt concentration when denitrification rate was zero (SA0) and salt concentration at the initiation of rate inhibition (SAI). Stress was 12.0.

Denitrifier community diversity in terms of richness, evenness, and phylogenetic diversity differed between the soil communities, as well as the community composition based on genotypic dissimilarties (Fig. 2) and phylogeny (Fig. 3). The 1152 *nosZ* clones from the 12 different libraries could be separated into 64 RFLP groups reflecting the genotypic dissimilarities. They were distributed among community A, B, C and J each with 30, 34, 23 and 44 groups, respectively (Figs. 2 and S2). Evenness also varied between communities, with C being dominated by only two RFLP groups (70% of all clones), while a more even distribution was observed in the other communities (Fig. S2). All communities exhibited unique RFLP groups: A with 3, B with 6, C with 7, and J with 12. Community J not only had the highest number of unique RFLP groups, but also shared the most RFLP groups with the other soil communities, while C shared the least (Fig. 2). Chao and *H’* indicated that communities A, B, and J had the highest diversity, while C had the lowest (Table 2). The AMOVA based on RFLP groups confirmed significant differences in denitrifier biodiversity among soil communities (*P*<0.001) and accounted for 17% of all the variation present in the dataset.

**Table 2:** Diversity indices of denitrifying bacterial communities determined from RFLP group membership using Chao and Shannon (*H*') metrics or by comparative sequence analysis for phylogenetic diversity (PD), net relatedness index (NRI), and nearest taxon index (NTI) based on *nosZ* gene clones from each soil community (n = 3).

Soil community (treatment)	Chao	*H'*	PD	NRI	NTI
A (Fallow)	34.2 (30.8–52.3)	2.73 (2.60–2.85)	5.61	10.18*	2.31*
B (Unfertilized)	49.2 (38.3–87.5)	2.54 (2.39–2.70)	6.67	−0.84	0.48
C (Nitratefertilized)	25.1 (23.3–36.4)	1.81 (1.66–1.97)	5.25	6.17*	3.47*
J (Cattle manurefertilized)	57.3 (48.1–87.6)	2.84 (2.69–3.00)	7.15	3.31*	1.30

Values in parentheses indicate the lower and upper 95% confidence intervals [27] and * indicate values significantly different from the null (*p*<0.05).

Phylogenetic diversity based on ML phylogenetic analyses of *nos*Z gene sequences (Fig. S3) revealed similar diversity patterns as the RFLP analysis. In the phylogeny, the RFLP-groups made up major clusters or sub-clusters within these, although the node support was too low to affiliate the RFLP-groups too specific clusters (Fig. S4). The PD metric indicated that J was the most diverse denitrifier community, followed by B, A and C (Table 2). Both NRI and NTI were positive and significant in A and C indicating a higher degree of phylogenetic clustering of the denitrifier community in these soils than would be expected by chance (Table 2). In pairwise correlation analyses, the RFLP group and phylogeny based diversity metrics Chao and PD, respectively were highly correlated (*r* ^2^ = 0.99, *P* = 0.009). The only diversity metric that correlated with parameters from the modeled denitrification rates was PD and optimum temperature (*T_m_*; *r* ^2^ = 0.53, *P* = 0.075).

Phylogeny-based community composition differences demonstrated in the NMS ordination of the distance matrix from the unweigthed UniFrac analysis showed clear separation of soil communities C and J, while communities A and B overlapped (Fig. 3). A Mantel test between the UniFrac matrix and a matrix of diversity metrics and modeled denitrification rate parameters for each community replicate (Tables S2,S3,S4) indicated a significant correlation (*r* = 0.62, *P* = 0.001). Vectors in the NMS indicate that diversity metrics and modeled rate parameters were significantly correlated with the ordination (*P*<0.1; Fig. 3). However, they were not directly supporting one another since vectors representing diversity metrics did not interact with the ordination in the same direction as modeled denitrification rate parameters.

## Discussion

Differences in the functional operating range were observed between four, naturally assembled soil bacterial communities when exposed to different environmental gradients. The functional operating range under both gradients tested was significantly broader in the soil community that was phylogenetically the most diverse (J) and harbored the highest number of total and unique community members. This supports that the insurance hypothesis, stating that biodiversity is important for ecosystem functioning under changing environmental conditions [12], is valid also for highly diverse and complex soil microbial communities performing specific functions. Nevertheless, the least diverse community (C), dominated by two RFLP groups and sharing the lowest number of community members with the other communities, displayed the same initial operating range as community J under the salt gradient. This indicates that specific, well adapted and high-functioning bacterial genotypes can play an important role for ecosystem functioning under certain, but not all conditions. Our results provide evidence that diversity and composition of microbial communities affect ecosystem functioning under fluctuating conditions and we suggest that the mechanism underpinning a broader operating range was enhanced complementarity due to increased genotypic dissimilarities [35] or selection for key genotypes [36].

The genotypic dissimilarities among the four soil communities are the result of 50 years of different fertilization regimes. These results are consistent with a large body of literature emphasizing the impact of agricultural practices on microbial communities, including nitrogen cycling communities and denitrifiers, through alteration of soil properties, e.g. [23], [37], [38]. Diversity metrics based on RFLP groups as well as phylogenetic diversity analyses indicated a higher diversity in community J, which was sampled from the cattle manure fertilized soil. This soil displayed carbon and nitrogen contents about twice as high as in the other soils in addition to the highest primary production (i. e. crop yield in Table S1). The latter is a frequently used proxy for resource availability expressed as the rate of organic matter production in a system. Thus, the observed higher diversity in community J is consistent with theory predicting that diversity is related to the availability of resources in the ecosystem [39]. Increased plant growth also results in increased carbon rich plant exudates that support bacterial growth [40]. The addition of manure in combination with increased primary productivity in the soil is expected to not only increase resource availability, but also resource complexity, which would support development of genetically diverse microbial communities [41], [42]. By contrast, community C that had developed under constant fertilization with mineral nitrogen had the lowest diversity for most diversity measures and was also the community displaying the lowest evenness. Similar patterns are known for plant and phytoplankton communities, which are driven to be dominated by a few species when fertilized with nitrogen (e.g. [43], [44]. Nevertheless, the effect of nitrogen may not be universal as others have found that nitrogen fertilization changes the soil bacterial community composition, but without affecting bacterial diversity [45]. Since nitrogen addition also stimulates primary production, one can assume that resource availability would have increased also for the microbial community present in the nitrogen fertilized soil. Indeed, both soil carbon and nitrogen content had increased compared to the unfertilized soil, but phosphorous and potassium concentrations were similar and even lower than in the unfertilized plots, and could have been limiting factors (Table S1). The significant positive NRI and NTI values for community C, but also A, indicate phylogenetically clustered denitrifier communities with less evolutionary diversity. Phylogenetic clustering has been attributed to habitat filtering or differential colonization abilities [32], [46]. In this study, colonization was probably not of importance since it is likely that the same denitrifier community was present across the field site before the establishment of the randomly distributed differently fertilized plots. Altogether, these results suggest a higher habitat filtering in community C than in the other communities indicating that the closely related genotypes share traits important for their persistence in this particular soil.

If we assume that distantly related genotypes co-exist due to less competition, increased phylogenetic dissimilarity should reflect increased functional dissimilarity. This has been shown true for plant, fungal and bacterial communities [8], [47], [48]. In agreement, the most diverse community (J), actually as verified by PD but also all other metrics, did have the broadest functional operating range for both the temperature and salt concentration gradient. However, we did not find a simple link between phylogenetic dissimilarity or any other diversity metric and the functional operating range of the corresponding microbial community. For instance, the least diverse soil community (C) had a similar range and optimum temperature as the intermediate diversity soil communities (A and B) under the temperature gradient, despite differences in primary productivity. Community C also coped with increasing salt concentration in a similar way as community J. Since not only the diversity but also the composition of the communities were modified by the fertilization regimes, it is likely that phenotypic trait variations in the communities are related to variations in stress tolerance physiologies. Depending on the individual responses and tolerances of the denitrifier community members, some community members were likely equally sensitive to both extreme temperature and salt conditions while others, like those in communities C and J, have a higher stress buffering capacity to extreme salt concentrations. The higher conductivity measured in soil C suggest that the community present in these nitrogen fertilized plots were adapted to higher salt concentrations and therefore initially sustained their denitrification capacity when salt concentrations increased. Due to their highly exergonic aerobic and anaerobic dissimilatory processes, denitrifiers can readily adapt to high salt concentrations by synthesizing osmotoic solutes [49]. Wittebolle et al. [9] found temperature or salt stresses to differentially affect the buffering capability of a microbial community and concluded that community evenness was more important than richness. However, our results indicate that community membership may play a more important role for soil functioning. This is supported by Ives and Carpenter [50], who suggested that depending on the type of perturbation, different species may be of importance for ecosystem stability. Community C exemplifies that ecosystem functioning can be maintained under certain, although not all, conditions when diversity is low due to filtering of key community members.

Our results do not establish a straightforward relationship between the functional operating range and a level of biodiversity, but rather show complex patterns that vary with the community composition and environmental conditions. Recent work has demonstrated that phylogenetic dissimilarity rather than species richness is positively linked with community functioning in microbial communities [8], [48]. However, in our study PD and relatedness metrics coincided with genotype richness. Although diversity was associated with the broadest functional operating range and the highest process rates, we demonstrated that community membership also played a crucial role for ecosystem functioning under certain conditions.

## Supporting Information

Figure S1 Denitrification rates at different temperatures and salt concentrations. The rates for each soil community replicate were modeled using a Gaussian function and a power equation for temperature (a) and salt concentration (b) gradients, respectively. The fitted curves and data points are colored by community and soil treatment: red, community A, fallow; green, community B, unfertilized; blue, community C, nitrate fertilized; and black, community J, cattle manure fertilized. Each field replicate within a treatment/community is represented by different symbols.
(DOCX)

Figure S2 Distribution of 1152 *nosZ* clones in the 64 RFLP groups detected among the soil communities. Community A had 30, community B had 34, community C had 23 and community J had 44 groups. Data are normalized by percent of total number of clones for each soil community.
(DOCX)

Figure S3 Maximum likelihood phylogenetic analysis of 400 *nosZ* gene sequences (708 bp) from soil communities. Community and soil treatment are colored: red, community A, fallow; green, community B, unfertilized; blue, community C, nitrate fertilized; and purple, community J, cattle manure fertilized. The tree was displayed and colored by treatment using the iTOL web-based tool.
(DOCX)

Figure S4 Maximum likelihood phylogenetic analysis of 100 *nosZ* gene sequences (713 bp) from soil clones selected to represent each of the 64 RFLP groups described in Fig. S2. Similar sequences from pure cultures and other soil clones retrieved from Genbank are included. RFLP group assignments are indicated in bold face. Taxonomic designations are listed on the right for Alphaproteobacteria (α), Gammaproteobacteria (γ), Betaproteobacteria (β). Boostrap values >70 are indicated at the nodes.
(DOCX)

Table S1 Soil properties for the Ultuna long-term soil organic matter experiment (mean±SD, n = 3). Values followed by the same letter indicate treatments without significant differences (*p*<0.05).
(PDF)

Table S2Goodness of fit and model parameters for denitrification rates in soil communities at different temperatures modeled for each field replicate using the following Gaussian equation: Denitrification rate =  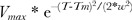 where 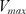 = maximum denitrification rate, T = tested temperature, 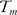 = optimum temperature and *w* = measure of the width of the curve. All model parameters were statistically significant (*p*<0.0007).(PDF)

Table S3 Goodness of fit and model parameters for denitrification rates in soil communities at different NaCl concentrations and modeled for each field replicate using the following power equation: Denitrification rate = *k*/√(*c*) - *a*, where *k* = slope, *c* = % salt concentration and *a* = intercept. The salt concentration when denitrification begins to be inhibited (SAI) and when the rate reaches zero (SA0) was extrapolated using the equation.
(PDF)

Table S4 Diversity indices of denitrifying bacterial communities determined using RFLP group membership for Chao and Shannons (*H’*) indices or by comparative sequence analysis for phylogenetic diversity (PD), net relatedness index (NRI), and nearest taxon index (NTI) based on *nosZ* gene clones from each soil community replicate.
(PDF)
