# Supplementary material for: Soil Functional Operating Range Linked to Microbial Biodiversity and Community Composition Using Denitrifiers as Model Guild
Source: PLoS One. 2012 Dec 20;7(12):e51962. doi: 10.1371/journal.pone.0051962 (PMC3527374; doi:10.1371/journal.pone.0051962)
Supplement: Figure S1 — Denitrification rates at different temperatures and salt concentrations. The rates for each soil community replicate were modeled using a Gaussian function and a power equation for temperature (a) and salt concentration (b) gradients, respectively. The fitted curves and data points are colored by community and soil treatment: red, community A, fallow; green, community B, unfertilized; blue, community C, nitrate fertilized; and black, community J, cattle manure fertilized. Each field replicate within a treatment/community is represented by different symbols. (DOCX) [file pone.0051962.s001.docx]

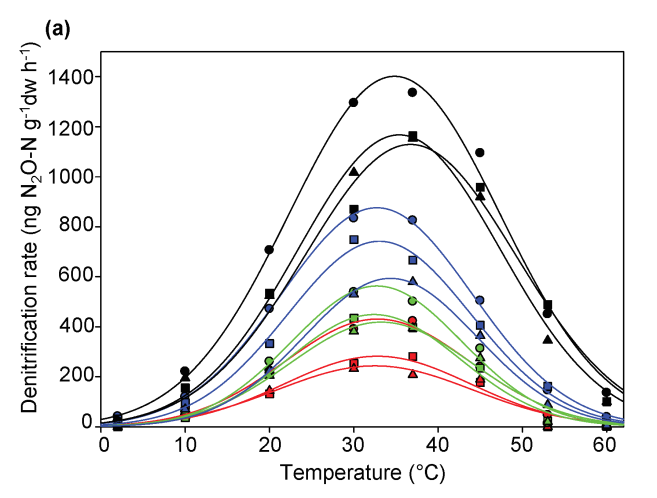

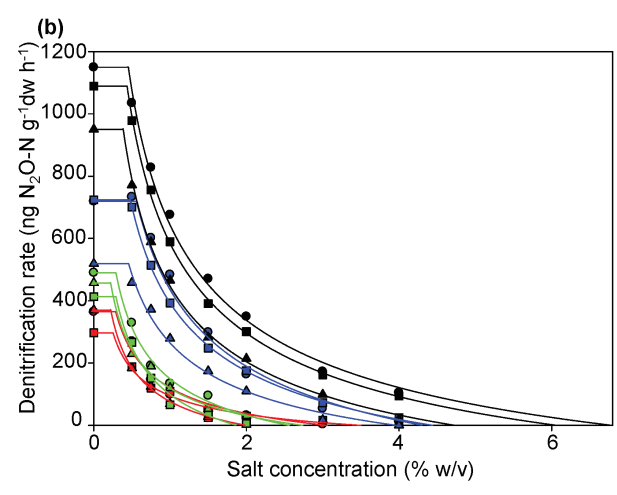


**Figure S1.** **Denitrification rates at different temperatures and salt concentrations.** The rates for each soil community replicate were modeled using a Gaussian function and a power equation for temperature (a) and salt concentration (b) gradients, respectively. The fitted curves and data points are colored by community and soil treatment: red, community A, fallow; green, community B, unfertilized; blue, community C, nitrate fertilized; and black, community J, cattle manure fertilized. Each field replicate within a treatment/community is represented by different symbols.
