# Supplementary material for: Soil Functional Operating Range Linked to Microbial Biodiversity and Community Composition Using Denitrifiers as Model Guild
Source: PLoS One. 2012 Dec 20;7(12):e51962. doi: 10.1371/journal.pone.0051962 (PMC3527374; doi:10.1371/journal.pone.0051962)
Supplement: Figure S2 — Distribution of 1152 nosZ clones in the 64 RFLP groups detected among the soil communities. Community A had 30, community B had 34, community C had 23 and community J had 44 groups. Data are normalized by percent of total number of clones for each soil community. (DOCX) [file pone.0051962.s002.docx]

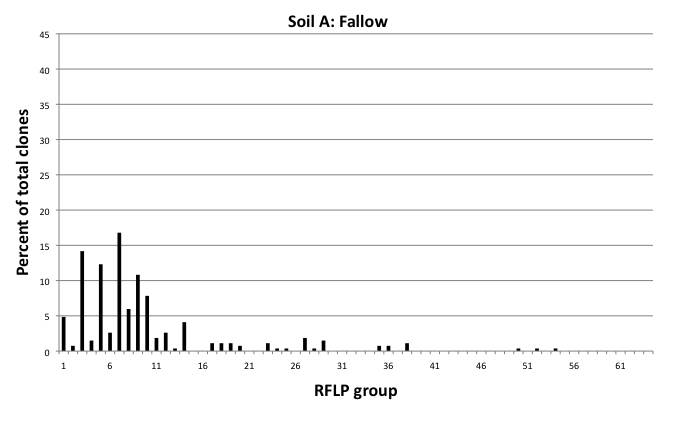
**
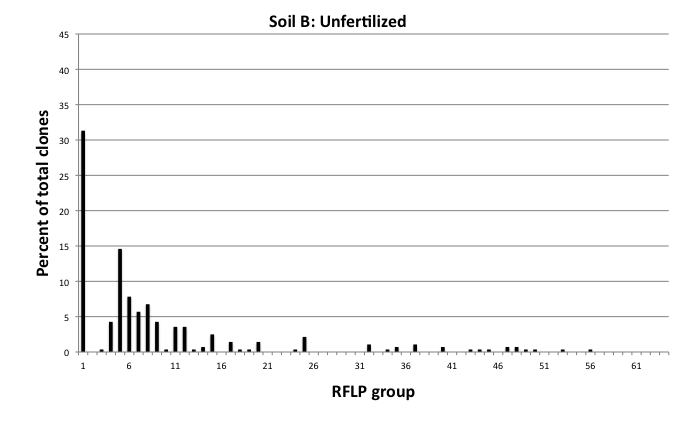
**


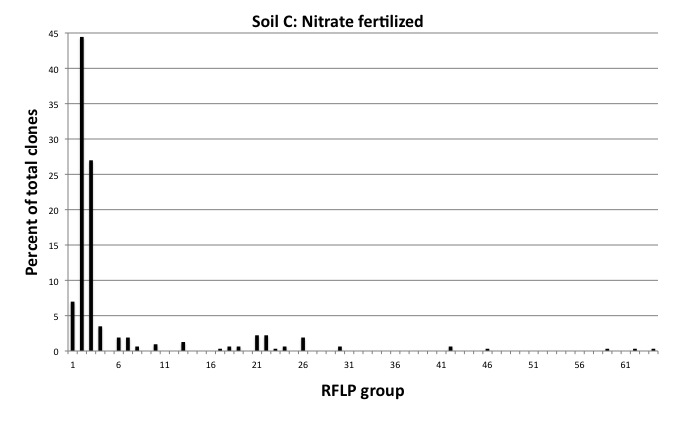

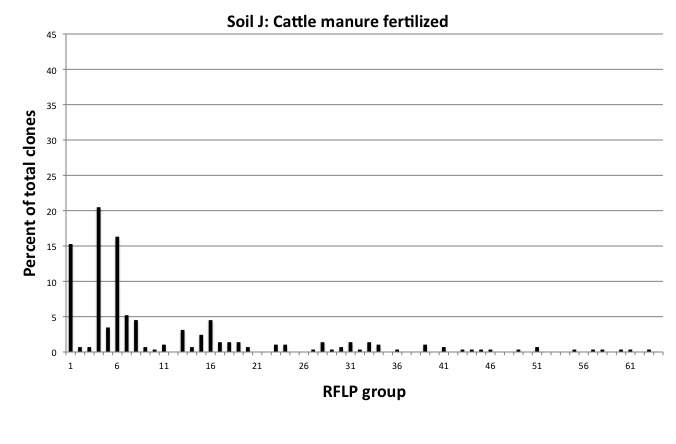


**Figure S2. Distribution of 1152 *nosZ* clones in the 64 RFLP groups detected among the soil communities.** Community A had 30, community B had 34, community C had 23 and community J had 44 groups. Data are normalized by percent of total number of clones for each soil community.
