# Supplementary material for: Soil Functional Operating Range Linked to Microbial Biodiversity and Community Composition Using Denitrifiers as Model Guild
Source: PLoS One. 2012 Dec 20;7(12):e51962. doi: 10.1371/journal.pone.0051962 (PMC3527374; doi:10.1371/journal.pone.0051962)
Supplement: Figure S3 — Maximum likelihood phylogenetic analysis of 400 nosZ gene sequences (708 bp) from soil communities. Community and soil treatment are colored: red, community A, fallow; green, community B, unfertilized; blue, community C, nitrate fertilized; and purple, community J, cattle manure fertilized. The tree was displayed and colored by treatment using the iTOL web-based tool. (DOCX) [file pone.0051962.s003.docx]

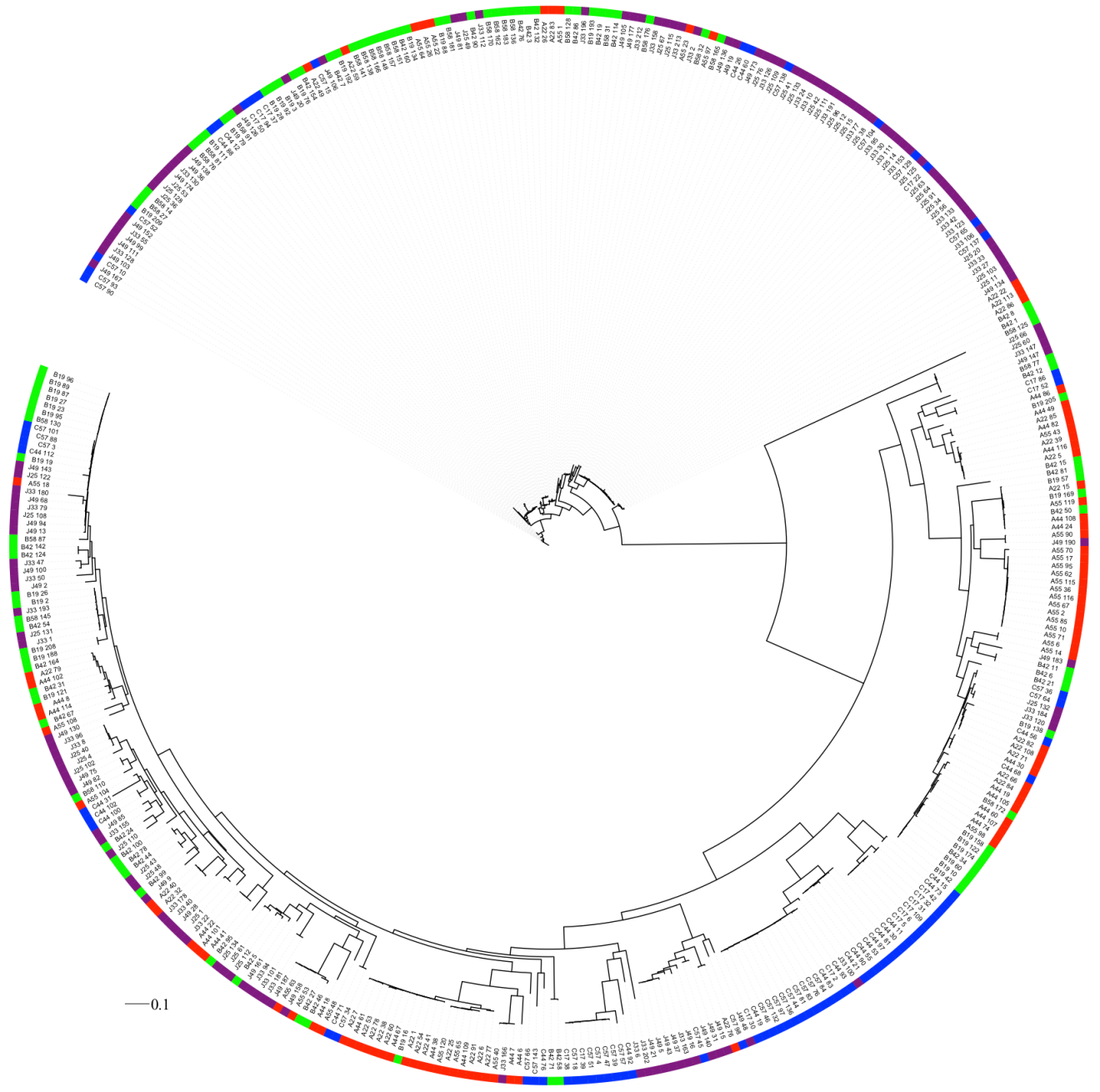


**Figure S3** **Maximum likelihood phylogenetic analysis of 400 *nosZ* gene sequences (708 bp) from soil communities colored by community and soil treatment**: red, community A, fallow; green, community B, unfertilized; blue, community C, nitrate fertilized; and purple, community J, cattle manure fertilized.. The tree was displayed and colored by treatment using the iTOL web-based tool.
