# Supplementary material for: Soil Functional Operating Range Linked to Microbial Biodiversity and Community Composition Using Denitrifiers as Model Guild
Source: PLoS One. 2012 Dec 20;7(12):e51962. doi: 10.1371/journal.pone.0051962 (PMC3527374; doi:10.1371/journal.pone.0051962)
Supplement: Figure S4 — Maximum likelihood phylogenetic analysis of 100 nosZ gene sequences (713 bp) from soil clones selected to represent each of the 64 RFLP groups described in Fig. S2. Similar sequences from pure cultures and other soil clones retrieved from Genbank are included. RFLP group assignments are indicated in bold face. Taxonomic designations are listed on the right for Alphaproteobacteria (α), Gammaproteobacteria (γ), Betaproteobacteria (β). Boostrap values >70 are indicated at the nodes. (DOCX) [file pone.0051962.s004.docx]

**Figure S4** **Maximum likelihood phylogenetic analysis of 100 *nosZ* gene sequences (713 bp) from soil clones selected to represent each of the 64 RFLP groups described in Fig. S2.** Similar sequences from pure cultures and other soil clones retrieved from Genbank are included. RFLP group assignments are indicated in bold face. Taxonomic designations are listed on the right for Alphaproteobacteria (α), Gammaproteobacteria (γ), Betaproteobacteria (β). Boostrap values >70 are indicated at the nodes.
