# Supplementary material for: Soil Functional Operating Range Linked to Microbial Biodiversity and Community Composition Using Denitrifiers as Model Guild
Source: PLoS One. 2012 Dec 20;7(12):e51962. doi: 10.1371/journal.pone.0051962 (PMC3527374; doi:10.1371/journal.pone.0051962)
Supplement: Table S1 — Soil properties for the Ultuna long-term soil organic matter experiment (mean±SD, n = 3). Values followed by the same letter indicate treatments without significant differences (p<0.05). (PDF) [file pone.0051962.s005.pdf]

Table S1. Soil properties for the Ultuna long-term soil organic matter experiment (mean±SD, n=3). Values followed by the same letter indicate treatments without significant differences (p<0.05)

Treatment	pH	Tot-N (%)	Tot-C (%)	C:N	P†	K†	Conductance (μS)	Crop yield§ (kg ha^{-1})
A:Fallow	6.2(0.2)[a]	0.10(0)[a]	0.9(0.02)[a]	9.7(0.1)[a]	21(0.6)[a]	27(2.0)[b]	59(3.6)[b]	NA
B:Unfertilized	6.2(0.1)[a]	0.11(0)[b]	1.1(0.03)[b]	9.9(0.1)[b]	17(1.6)[a]	21(2.3)[ab]	43(1.2)[a]	1288(351)[a]
C:Nitrate fertilized	6.6(0.3)[a]	0.13(0)[c]	1.4(0.06)[c]	10.3(0.1)[c]	17(2.7)[a]	16(1.0)[a]	74(3.4)[c]	3604(791)[ab]
J:Cattle manure	6.5(0.2)[a]	0.20(0)[d]	2.1(0.06)[d]	10.5(0.1)[d]	37(2.7)[b]	49(1.0)[c]	52(4.5)[ab]	4775(1823)[b]

When the experimental site was established in 1956, the soil pH was 6.5 and the Tot-C and Tot-N were 1.5% and 0.17% of the soil dry weight (dw).
† Ammonium acetate lactate dissolved P or K in the topsoil (mg^{-1} 100g air dried soil).
§ Total dry weight of green biomass.
