# Supplementary material for: Soil Functional Operating Range Linked to Microbial Biodiversity and Community Composition Using Denitrifiers as Model Guild
Source: PLoS One. 2012 Dec 20;7(12):e51962. doi: 10.1371/journal.pone.0051962 (PMC3527374; doi:10.1371/journal.pone.0051962)
Supplement: Table S2 — Goodness of fit and model parameters for denitrification rates in soil communities at different temperatures modeled for each field replicate using the following Gaussian equation: Denitrification rate = where = maximum denitrification rate, T = tested temperature, = optimum temperature and w = measure of the width of the curve. All model parameters were statistically significant (p<0.0007). (PDF) [file pone.0051962.s006.pdf]

Table S2. Goodness of fit and model parameters for denitrification rates in soil communities at different temperatures modeled for each field replicate using the following Gaussian equation: Denitrification rate = $V_{max} * e^{-(T-Tm)^2/(2*w^2)}$ where V_{max} = maximum denitrification rate, T = tested temperature, T_m = optimum temperature and w = measure of the width of the curve. All model parameters were statistically significant (p<0.0007).

Soil community replicate	Adj r^2†	V_{max}	w	T_m
A1	0.981	431	11.0	32.8
A2	0.879	282	11.1	32.8
A3	0.881	244	11.6	32.5
B1	0.976	563	10.1	32.7
B2	0.986	449	10.2	32.4
B3	0.952	419	10.8	33.2
C1	0.996	876	11.3	32.7
C2	0.989	742	10.9	33.0
C3	0.989	594	10.3	34.3
J1	0.989	1402	12.5	34.8
J2	0.965	1129	12.5	36.7
J3	0.983	1167	12.0	35.4

† Adjusted r^2 reflecting the goodness of fit for the model.
