# Supplementary material for: Soil Functional Operating Range Linked to Microbial Biodiversity and Community Composition Using Denitrifiers as Model Guild
Source: PLoS One. 2012 Dec 20;7(12):e51962. doi: 10.1371/journal.pone.0051962 (PMC3527374; doi:10.1371/journal.pone.0051962)
Supplement: Table S3 — Goodness of fit and model parameters for denitrification rates in soil communities at different NaCl concentrations and modeled for each field replicate using the following power equation: Denitrification rate = k/√(c) - a, where k = slope, c = % salt concentration and a = intercept. The salt concentration when denitrification begins to be inhibited (SAI) and when the rate reaches zero (SA0) was extrapolated using the equation. (PDF) [file pone.0051962.s007.pdf]

Table S3. Goodness of fit and model parameters for denitrification rates in soil communities at different NaCl concentrations and modeled for each field replicate using the following power equation: Denitrification rate = $k/\sqrt{(c)} - a$, where k = slope, c = % salt concentration and a = intercept. The salt concentration when denitrification begins to be inhibited (SAI) and when the rate reaches zero (SA0) was extrapolated using the equation.

Soil community replicate	r^2†	k	a	SAI§	SA0‡
A1	0.938	282	161	0.29	3.06
A2	0.941	204	109	0.25	3.51
A3	0.994	262	186	0.22	2.00
B1	0.977	398	248	0.29	2.57
B2	0.964	368	269	0.29	1.87
B3	0.970	300	181	0.22	2.74
C1	0.983	840	404	0.56	4.32
C2	1.000	747	355	0.48	4.44
C3	0.989	531	267	0.46	3.97
J1	0.993	1044	400	0.45	6.79
J2	0.999	983	399	0.44	6.07
J3	0.997	827	380	0.39	4.74

† r^2 reflecting the goodness of fit for the model to the denitrification rate data.
§ Salt concentration when denitrification inhibition begins (SAI), calculated from the model.
‡ Salt concentration when the denitrification rate reach zero (SA0), extrapolated from the model.
