# Supplementary material for: Soil Functional Operating Range Linked to Microbial Biodiversity and Community Composition Using Denitrifiers as Model Guild
Source: PLoS One. 2012 Dec 20;7(12):e51962. doi: 10.1371/journal.pone.0051962 (PMC3527374; doi:10.1371/journal.pone.0051962)
Supplement: Table S4 — Diversity indices of denitrifying bacterial communities determined using RFLP group membership for Chao and Shannons (H’) indices or by comparative sequence analysis for phylogenetic diversity (PD), net relatedness index (NRI), and nearest taxon index (NTI) based on nosZ gene clones from each soil community replicate. (PDF) [file pone.0051962.s008.pdf]

Table S4. Diversity indices of denitrifying bacterial communities determined using RFLP group membership for Chao and Shannons (*H'*) indices or by comparative sequence analysis for phylogenetic diversity (PD), net relatedness index (NRI), and nearest taxon index (NTI) based on *nosZ* gene clones from each soil community replicate.

Soil community replicate	Chao	*H'*	PD	NRI	NTI
A1	17.00 (16.11-24.97)	2.34 (2.16-2.53)	3.57	3.35*	2.91*
A2	25.00 (19.34-54.54)	2.33 (2.14-2.53)	3.77	9.12*	2.57*
A3	28.00 (22.34-57.54)	2.58 (2.38-2.79)	4.35	3.85*	1.18
B1	26.00 (20.34-55.54)	2.38 (2.15-2.61)	3.32	0.69	2.91*
B2	46.00 (29.58-110.73)	2.68 (2.49-2.87)	4.68	0.32	1.57*
B3	18.00 (13.78-45.14)	1.71 (1.48-1.95)	4.20	5.44*	-0.05
C1	10.00 (9.09-19.68)	1.19 (0.96-1.42)	3.00	1.53	3.41*
C2	20.50 (14.32-55.53)	1.69 (1.45-1.93)	4.08	5.11*	2.08*
C3	23.00 (17.34-52.54)	1.63 (1.37-1.90)	3.97	1.99*	2.68*
J1	27.00 (22.33-48.10)	2.46 (2.23-2.70)	3.56	4.16*	3.05*
J2	39.00 (27.50-88.24)	2.65 (2.43-2.86)	5.10	1.44	0.26
J3	38.67 (32.37-61.66)	2.75 (2.53-2.98)	4.99	-0.04	0.60

Values in parentheses indicate the lower and upper 95% confidence intervals and *indicate values significantly different from the null (p<0.05; Oksanen *et al.* 2011).

References

Oksanen, J., Blanchet, F.G., Kindt, R., Legendre, P., O'Hara, R.B., Simpson, G.L., Solymos, P., Stevens, M.H.H. & Wagner, H. (2011). vegan: community ecology package, v. 1.17-78 (Available at: http://vegan.r-forge.r-project.org/). Accessed January 15, 2011.
